# Uptake and translocation mechanisms of different forms of organic selenium in rice (*Oryza sativa* L.)

**DOI:** 10.3389/fpls.2022.970480

**Published:** 2022-08-22

**Authors:** Qi Wang, Lingxuan Kong, Qingqing Huang, Huafen Li, Yanan Wan

**Affiliations:** ^1^Beijing Key Laboratory of Farmland Soil Pollution Prevention and Remediation, Key Laboratory of Plant-Soil Interactions of the Ministry of Education, China Agricultural University, Beijing, China; ^2^Innovation Team of Remediation of Heavy Metal-Contaminated Farmlands, Agro-Environmental Protection Institute, Ministry of Agriculture and Rural Affairs, Tianjin, China

**Keywords:** rice, selenomethionine, selenomethionine-oxide, uptake kinetics, transport, interaction

## Abstract

Selenium (Se) is an essential trace element for human and animal health, and toward an understanding of the uptake and translocation of Se in plants is important from the perspective of Se biofortification. In this study, we conducted hydroponic experiments to investigate the mechanisms of organic Se [selenomethionine (SeMet) and selenomethionine-oxide (SeOMet)] uptake, translocation, and the interactions between SeMet and SeOMet in rice. We also investigated differences in the dynamics of organic and inorganic Se uptake by rice roots. Concentration-dependent kinetic results revealed that SeMet uptake during a 1 h exposure was 3.19–16.0 times higher than that of three other Se chemical forms, with uptake capacity (*V_max_*) values ordered as follows: SeMet>SeOMet>selenite>selenate. Furthermore, time-dependent kinetic analysis revealed that SeMet uptake by roots and content in shoots were initially clearly higher than those of SeOMet, although the differences gradually diminished with prolonged exposure time; while no significant difference was found in the transfer factor of Se from rice roots to shoots between SeMet and SeOMet. Root uptake of SeOMet was significantly inhibited by carbonyl cyanide 3-chlorophenylhydrazone (CCCP) (30.4%), AgNO_3_ (41.8%), and tetraethylammonium chloride (TEACl) (45.6%), indicating that SeOMet uptake is a metabolically active process, and that it could be mediated *via* aquaporins and K^+^ channels. Contrarily, SeMet uptake was insensitive to CCCP, although markedly inhibited by AgNO_3_ (93.1%), indicating that rice absorbs SeMet primarily *via* aquaporins. Furthermore, Se uptake and translocation in rice treated simultaneously with both SeMet and SeOMet were considerably lower than those in rice treated with SeMet treatment alone and notably lower than the theoretical quantity, indicating interactions between SeMet and SeOMet. Our findings provide important insights into the mechanisms underlying the uptake and translocation of organic Se within plants.

## Introduction

Selenium (Se), an essential micronutrient with respect to human and animal health, is a necessary component of more than 30 Se-containing proteins and enzymes in mammals and is associated with multiple properties, including antioxidative, immunological, and anticarcinogenic effects (Rayman, 2012; Avery and Hoffmann, 2018). Indeed, a deficiency in Se can lead to the risk of cardiovascular and cancer diseases (Hatfield et al., 2014). Although the World Health Organization (WHO) recommends a daily Se intake of 50–200 μg d^–1^ for adults, given marked differences in the soil contents of Se worldwide and the distribution of Se-poor soils in some notably populous regions, Se deficiency may afflict as many as one billion people globally (Combs, 2001; Haug et al., 2007).

Selenium has multiple beneficial effects on the plant growth at appropriate concentration, including the enhancement of antioxidant capacity and photosynthesis (Jiang et al., 2021; Lanza et al., 2021a); while excessive Se can be detrimental (Lanza et al., 2021b). Concerning its beneficial effects on plant growth and human health, plants can be strategically utilized to regulate the effects of Se on both. Consumption of Se-rich plant food is considered to be the most effective approach to increase human Se uptake (White and Broadley, 2009; Alfthan et al., 2015), and accordingly, in those regions characterized by Se-deficient soils, agronomic biofortification based on Se fertilization could be practiced to produce Se-rich crops, thereby enhancing human Se intake.

Among different factors influencing the accumulation of Se in plants, the uptake and translocation of this element are the most fundamental physiological aspects. Consequently, gaining an in-depth understanding of the associated processes and mechanisms is important from the perspective of developing Se biofortification strategies. In natural environment, a range of distinct chemical forms of Se exists, and among which, selenite (SeIV) and selenate (SeVI) are the two most abundant forms in soil, with selenite predominating in soils characterized by intermediate redox potentials and selenate predominating under aerobic and neutral to alkaline conditions (Elrashidi et al., 1987). Although both selenate and selenite can be absorbed from soil by plant roots, it appears that neither is taken up *via* Se-specific transporters. Selenate is typically taken up *via* sulfate transporters (Sors et al., 2005; Mehdawi et al., 2018), and selenite might be absorbed *via* silicon influx transporters (Zhao et al., 2010) or incorporated into an active process mediated by phosphate transporters (Li et al., 2008; Zhang et al., 2014). Upon uptake, most selenite is rapidly metabolized to organic Se compounds and retained within the root system, whereas selenate can be rapidly translocated to the shoots (Li et al., 2008; Huang et al., 2017; Gong et al., 2018; Yu et al., 2019). In some soils, Se is also present as organic forms, such as SeMet and SeCys (Kikkert and Berkelaar, 2013); however, compared with those of the inorganic forms of Se, the uptake mechanisms of organic forms of Se is less investigated.

Rice (*Oryza sativa* L.) is a staple food for nearly half of the world’s population and one of major sources of the dietary Se intake (Rayman et al., 2008). In this regard, in addition to the total concentration of Se in crops, Se speciation is of particular importance in terms of its different health benefits (Zhu et al., 2009). Research has shown that in the mature grains of rice, Se is present primarily in organic forms, and among which, selenomethionine (SeMet) predominates (Sun et al., 2010; Huang et al., 2018). SeMet can be incorporated into proteins either directly or non-specifically *via* the replacement of methionine and is thereby readily absorbed by humans (Fairweather-Tait et al., 2010). Moreover, the uptake rate of SeMet by plants is higher than that of either selenite or selenate (Kowalska et al., 2020; Wang M. K. et al., 2020). Selenomethionine-oxide (SeOMet) is a derivative obtained from the transformation of SeMet. In our previous study, we detected SeOMet in the soil solution extracted from natural and selenite-supplied soils (Li et al., 2010), and SeOMet was also found in plants growing in media supplemented with either selenite or SeMet (Li et al., 2008; Kowalska et al., 2020). However, the uptake of SeOMet in plant roots remain unclear, and differences between organic and inorganic Se uptake are insufficiently well documented. Since SeMet and SeOMet exist in the forms of uncharged molecules, we speculate that the uptake of these two Se forms might be mediated by aquaporins, with high uptake potential in plant roots. In addition, when supplied with different chemical forms of Se simultaneously, a non-additive effect on the uptake would occur, for example, the presence of selenite appeared to inhibit the uptake of selenate in plants (Li et al., 2008; Wang et al., 2019). Thus we speculate a certain interaction might occur between SeMet and SeOMet during uptake process. To gain further insights in these regards, we conducted a series of hydroponic experiments to investigate (1) differences between the dynamics of organic and inorganic Se uptake by rice roots, (2) the mechanisms associated with the uptake of SeMet and SeOMet by roots and their subsequent translocation in plant, and (3) the interactions between SeMet and SeOMet during the uptake and translocation processes. We anticipated that the findings of this study would provide a theoretical basis for increasing Se levels and accumulating more organic Se in crops.

## Materials and methods

### Plant culture

For the purposes of this study, we used the rice (*Oryza sativa* L.) cultivar Zhuliangyou120 (a common *indica*-type cultivar). Rice seeds were surface sterilized with 30% (v/v) H_2_O_2_ for 15 min, rinsed thoroughly with deionized water, soaked in saturated CaSO_4_ solution in the dark overnight, and then germinated in a 0.5 mM CaCl_2_ solution. Seven days after germination, rice plants were transplanted into plastic pots containing 3 L of modified ½ Kimura nutrient solution (Huang et al., 2015), with the following composition (mmol L^–1^): KNO_3_ 0.091, KH_2_PO_4_ 0.1, Ca (NO_3_)_2_⋅4H_2_O 0.183, MgSO_4_⋅7H_2_O 0.274, (NH_4_)_2_SO_4_ 0.183, Fe (e1-EDTA 6.0 × 10^–2^, ZnSO_4_⋅7H_2_O 1.0 × 10^–3^, H_3_BO_3_ 3.0 × 10^–3^, (NH_4_)_6_Mo_7_O_24_⋅4H_2_O 1.0 × 10^–3^, MnSO_4_⋅H_2_O 1.0 × 10^–3^ and CuSO_4_⋅5H_2_O 2.0 × 10^–4^. The pH of the solution was buffered with 2 mM 2-morpholinoethanesulfonic acid (MES) and adjusted to a value of 5.5 with either 1 mM KOH or HCl. The solution was renewed at 3-day intervals.

Hydroponic experiments were conducted on a bench within a greenhouse of the China Agricultural University, Beijing. The conditions of the growth environment was as follows: day/night temperatures of 30 ± 2°C/23 ± 2°C; a light period of 14 h, with illumination provided by natural sunlight supplemented with sodium vapor lamps to maintain a light intensity of 240–350 μmol m^–2^ s^–1^; and a relative humidity of 60–70%.

### Selenium sources

Selenite and selenate were obtained from Sigma-Aldrich (St Louis, MO, United States), SeMet was provided by Shanxi University, and SeOMet was prepared by reacting SeMet with 3% H_2_O_2_ under sonication for 1 h (Larsen et al., 2004).

### Concentration-dependent kinetics of Se uptake

To evaluate the uptake capacities of rice roots with respect to organic and inorganic Se, we transferred 4-week-old rice plants to 250 mL of Se uptake solutions, each containing different chemical forms of Se, namely, selenite, selenate, SeMet, and SeOMet. For each of the different uptake solutions, a series of Se treatment solution were prepared with the Se concentration ranging from 0 to 20 μM (0, 0.1, 0.5, 1, 5, 10, and 20 μM at pH 5.5). Each treatment had four replicates with one plant per replicate. After 1 h of uptake incubation, rice roots were rinsed three times with deionized water and then transferred to 150-mL of ice-cold desorption solution (1 mM CaSO_4_ + 2 mM MES, pH 5.5) for 15 min to remove the Se adsorbed on root surfaces (Li et al., 2008). Following desorption, the rice roots were rinsed three times in deionized water and thereafter separated from the shoots. The root samples were oven-dried at 105°C for 30 min and 75n for 48 h, after which, they were weighed and used for determinations of Se concentrations.

### Time-dependent kinetics of selenomethionine and selenomethionine-oxide uptake

This experiment was conducted to investigate the temporal patterns of organic Se uptake and translocation by rice. Four-week-old rice plants were transferred to 1-L plastic container (one plant per pot) containing uptake solutions [with normal nutrients (control) and supplemented with either 5 μM SeMet or 5 μM SeOMet (2 mM MES, pH 5.5)], to which they were exposed for 1, 3, 5, 18, 26, 48, or 72 h, a control treatment (without any Se) was also conducted. Each treatment had four replicates. Following organic Se absorption, the roots were rinsed with deionized water and desorbed as described previously. The roots and shoots were then oven-dried and analyzed for Se concentrations.

### Effects of inhibitors on the uptake of selenomethionine and selenomethionine-oxide

To investigate the physiological processes and mechanisms of organic Se uptake, we examined the effects of the following inhibitors on the uptake of Se by rice: AgNO_3_, CoCl_2_, tetraethylammonium chloride (TEACl), 4,4-diisothiocyanatostilbene-2,2-disulfonic acid disodium salt hydrate (DIDS), and carbonyl cyanide 3-chlorophenylhydrazone (CCCP). AgNO_3_ is an aquaporin inhibitor that inhibits the water permeability of root cell plasma membranes (Niemietz and Tyerman, 2002), whereas CoCl_2_, TEACl, and DIDS act as inhibitors of Ca^2+^ (Harada and Shimazaki, 2009), K^+^ (White, 1995), and anion channels (Zhang et al., 2013), respectively, and the protonophore CCCP is a metabolic inhibitor. All inhibitors used in the study were obtained from Sigma-Aldrich (St Louis, MO, United States).

Four-week-old plants were transferred to uptake solutions containing 5 μM organic Se (SeMet or SeOMet) and different inhibitors (100 μM AgNO_3_, 5 mM TEACl, 5 mM CoCl_2_, 100 μM DIDS, or 1 μM CCCP, respectively), a control treatment (without any Se) was also conducted. CCCP was dissolved in ethanol and added to the solution at a final concentration of 0.01% (v/v) (Li et al., 2008). Consequently, we also included an additional control treatment containing 0.01% (v/v) ethanol. Four replicates were used for each treatment. After exposure for 1 h, the treated roots were rinsed with deionized water and the Se adsorbed on root surfaces was desorbed as described previously. Thereafter, the roots were oven-dried and analyzed for Se concentrations.

### Effects of P or S starvation on selenomethionine and selenomethionine-oxide uptake and translocation

This experiment was conducted to investigate whether phosphorus (P) or sulfur (S) starvation would influence the uptake and translocation of organic Se by rice. Four-week-old plants were transferred to 1-L plastic containers and treated with normal, P-deficient, or S-deficient nutrient solutions for 7 days. In the P-deficient and S-deficient solution, MgSO_4_, ZnSO_4_, KH_2_PO_4_, or CuSO_4_ were replaced by the corresponding chloride salts. At the end of the treatment period, the plants were transferred to a normal nutrient solution (modified **½** Kimura nutrient solution), to which either 5 μM SeMet or 5 μM SeOMet was added, followed by incubation for a further 2 days, a control treatment (without any Se) was also conducted. Then, the roots were desorbed, rinsed, oven-dried, and analyzed for Se concentrations. Each treatment had four replicates.

### Interactions between selenomethionine and selenomethionine-oxide

In this experiment, we sought to characterize the interactions between SeMet and SeOMet during their uptake and translocation in rice plants. Four-week-old plants were transferred to 250-mL containers, containing one of three different absorption solutions with the same total Se concentration (5 μM SeMet, 5 μM SeOMet, or 2.5 μM of both SeMet and SeOMet), a control treatment (without any Se) was also conducted. Each treatment had four replicates. After exposure for 1 h, the rice roots were rinsed three times with deionized water and desorbed as described previously. Thereafter, the roots and shoots were harvested, washed, oven-dried, and analyzed for Se concentrations.

### Analysis of Se content

For Se content analyses, 0.2500 g dried root and shoot samples were digested with 8 mL HNO_3_ (Guaranteed reagent) using a CEM MARS5 microwave sample preparation system (CEM Corp., Matthews, NC, United States). A 4 mL volume of the digest solution was then mixed with 1 mL of 6 M HCl and heated at 95 for 2 h to reduce selenate to selenite. Concentrations of Se in the mixed solution were determined by atomic fluorescence spectrometry using an AFS-920 Dual-channel Atomic Fluorescence Spectrometer, (Beijing Jitian Instruments Co., Ltd., Beijing, China). For quality assurance, we simultaneously analyzed a certified reference material (GBW10014, cabbage) and blanks, the recovery of Se in GBW10014 was 85–110%.

### Data analysis

Se uptake kinetics were described based on the Michaelis–Menten Equation:


$$V=\frac{V_{\text{max}}\times C}{K_{\text{m}}+C}$$

where *V* represents the uptake rate [μg g^–1^ root DW (dry weight) h^–1^], *K*_*m*_ represents the Michaelis constant (μM), *V*_*max*_ represents the maximal uptake rate (μg g^–1^ root DW h^–1^), and *C* represents the substrate concentration (μM). The Michaelis–Menten equation is of particular value with respect to the evaluation of transporter-mediated uptake processes. Uptake capacity (*V*_*max*_) is the maximal transport rate when all available carrier sites are saturated, whereas substrate affinity (*K*_*m*_) is equal to the substrate concentration at which the reaction rate is half-maximal.

All results are expressed as mean values with corresponding standard errors (*n* = 4). Two-way analysis of variance (ANOVA) was performed to test the significance of the Se form, treatment time (or inhibitor type, nutrient status) and the interactions between them, by using SAS 9.3 statistical software with least significant difference (LSD, *P* < 0.05).

## Results

### Concentration-dependent kinetics of Se uptake

**FIGURE 1:**
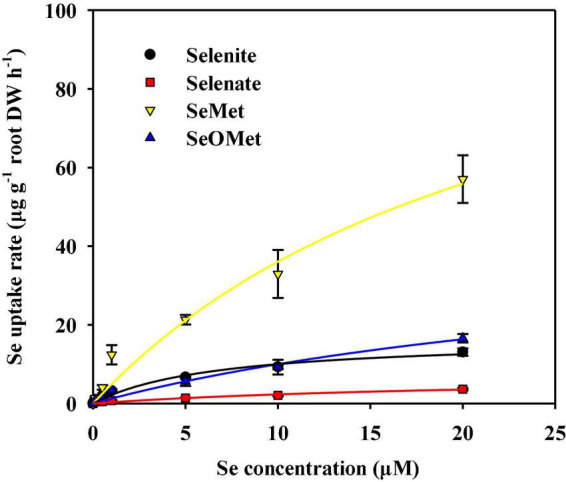
Concentration-dependent kinetics for the influx of different forms of Se into rice roots within 1 h. Data are presented as mean ± SE (*n* = 4). The curves represent the fitted Michaelis–Menten kinetics.

We found that uptake of the four assessed Se chemical forms by rice roots increased concomitant with an increase in the Se concentration of the uptake solution, all of which were satisfactorily described by the Michaelis–Menten equation (Figure 1 and Table 1). With the exception of SeMet, the influx of the remaining three chemical forms of Se into roots had features of saturating kinetics within the concentration range from 0 to 20 μM (Figure 1). The Michaelis–Menten kinetics curves also showed that, in the uptake solution containing 5–20 μM Se, the rate of SeMet uptake was 3.19–16.0 times higher than that of the other three Se chemical forms. In addition, we established that the chemical forms of Se had a marked effect (*P* < 0.05) on *V*_*max*_ and *K*_*m*_ values (Table 1). The calculated values of *V*_*max*_ declined in the order of SeMet > SeOMet > selenite > selenate, thereby indicating that organic forms of Se, particularly SeMet, are characterized by a considerably higher uptake potential in rice roots than inorganic forms. Moreover, we found that the *K*_*m*_ values calculated for selenate, SeMet, and SeOMet uptake were 2.42-, 2.64-, and 4.18-fold higher than those of selenite, respectively, indicating that rice roots have a higher affinity for selenite than for the other assessed Se chemical forms. Interestingly, we observed that the absorption kinetics of selenite and SeOMet in rice differed according to exogenous Se concentrations. Specifically, at Se concentrations between 0 and 10 μM, the rate of selenite uptake was higher than that of SeOMet (*P* > 0.05), whereas the opposite response (selenite < SeOMet) was detected at higher Se concentrations ranging from 10 to 20 μM (*P* > 0.05), and the two corresponding Michaelis–Menten curves had a single point of intersection at 10 μM (Figure 1).

**TABLE 1:** Kinetic parameters for the influx of four forms of Se into rice roots.

Treatment	*V*_*max*_ (μg⋅g^–1^ root DW h^–1^)	*K*_*m*_ (μM)	*R*
Selenite	16.9 ± 2.16	6.67 ± 2.20	0.988***
Selenate	7.67 ± 3.24	22.8 ± 15.7	0.973**
SeMet	125 ± 42.2	24.3 ± 13.0	0.986***
SeOMet	45.1 ± 20.7	34.6 ± 22.9	0.985***

***p < 0.001; **P < 0.01.

### Time-dependent kinetics of organic Se uptake and translocation

**FIGURE 2:**
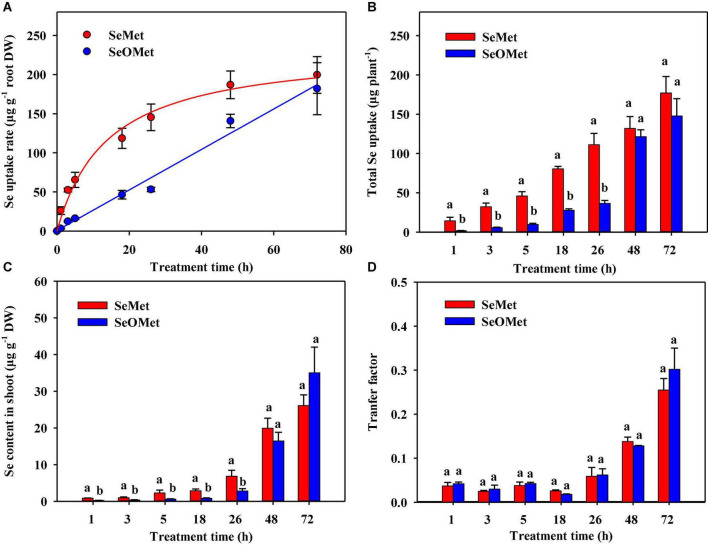
Time-dependent kinetics of the uptake of organic Se by rice roots **(A)**, total Se uptake **(B)**, the contents of Se in rice shoots **(C)**, and the transfer factor of Se from roots to shoots **(D)**. Data are presented as mean ± SE (*n* = 4). Different lowercase letters above bars indicate significant differences between SeMet and SeOMet treatments in individual treatment times (*P* < 0.05).

Our time-dependent analysis of organic Se uptake revealed it was significantly affected by Se form, treatment time, and their interaction between these two factors (*P* < 0.001). And the Se in rice plants in the control treatment was below the detection level (the same below). Generally, the uptake rate of Se as SeMet was higher than that of the SeOMet form at all assessed time points, although the differences narrowed with time. For example, although SeMet uptake rate after exposure for up to 26 h was 1.56–7.19-fold higher than that of SeOMet, however, after exposure for 48 h, the uptake rate of Se as SeMet was only 9.60–32.8% higher (Figure 2A). Furthermore, irrespective of the chemical forms, the uptake rate of Se increased with a prolongation of treatment time, although the increase in SeMet uptake rate declined with time and that of SeOMet remained relatively constant. In neither case, however, did the rate of Se uptake reach a plateau during the assessed treatment period. A similar tendency was obtained with respect to the total Se uptake: within 26 h, the total Se in SeMet treatments was 1.89–7.09-fold higher than that of SeOMet treatments; while the difference was only 8.82–19.8% after 48 h (Figure 2B).

When assessed at time points prior to 26 h, the contents of Se in the shoots of plants treated with SeOMet were between 58.6 and 72.3% lower than those in the plants treated with SeMet. However, in response to prolonged exposure for 48 and 72 h, the differences between these two Se chemical forms became non-significant (Figure 2C). In order to evaluate the root-to-shoot translocation ability of different Se sources, the transfer factor was introduced in the study. In general, irrespective of the Se treatments, the transfer factor of Se from rice roots to shoots showed an increasing trend with increase of the exposure time, varied from 0.025 to 0.302; while the lowest transfer factor was appeared at 18 h. In addition, there was no significant difference of the transfer factor between SeMet and SeOMet treatments at all exposure time (Figure 2D).

### Effects of inhibitors on the uptake rate of organic Se

**FIGURE 3:**
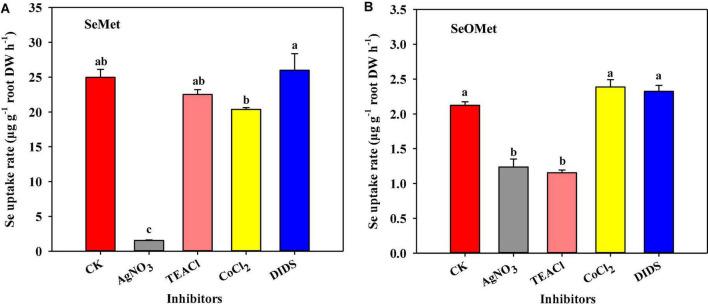
Effect of different specific inhibitors on the uptake of Se by rice supplied with SeMet **(A)** and SeOMet **(B)**. Data are presented as mean + SE (*n* = 4). Different lowercase letters above bars indicate significant differences among the inhibitor treatments (*P* < 0.05).

To gain further insights into the uptake process, we examined the effects of exposure to four specific inhibitors on uptake of the two assessed chemical forms of organic Se following 1 h exposures. Our observations revealed that Se uptake rate was significantly affected by Se form, inhibitor type, and their interaction between these two factors (Figure 3, *P* < 0.001). Generally, the uptake of Se as SeMet by rice roots was substantially higher than that as SeOMet, the former being 1.25–18.5-fold higher than the latter. Compared with the control, the exposure to AgNO_3_ significantly reduced the uptake of Se as SeMet and SeOMet by 93.1 and 41.8%, respectively (*P* < 0.05); whereas, although TEACl significantly inhibited the uptake of Se as SeOMet by 45.7% (*P* < 0.05), no significant inhibitory effect was detected in the uptake of SeMet. Contrastingly, neither CoCl_2_ nor DIDS had any significant inhibitory effects on Se uptake as either SeMet or SeOMet.

**FIGURE 4:**
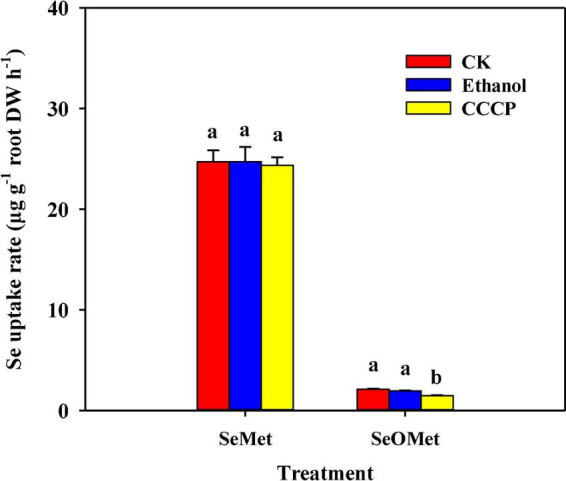
Effect of the respiratory inhibitor CCCP on the uptake of Se by rice supplied with different forms of organic Se. Data are presented as mean + SE (*n* = 4). Different lowercase letters above bars indicate significant differences among inhibitor treatments in individual Se treatment forms (*P* < 0.05).

Moreover, whereas the exposure to the metabolic inhibitor CCCP had no significant effect on the uptake of Se as SeMet, a significant reduction of 30.4% was detected for SeOMet form. The presence of ethanol in this treatment had no significant effect on Se uptake (Figure 4).

### Effects of P or S starvation on organic Se uptake and translocation

This experiment was designed to determine the effects of major macronutrients P and S on the uptake and translocation of different organic chemical forms of Se. Rice plants were grown in normal, S-deficient, or P-deficient medium for 7 days, after which, they were transferred to media containing either 5 μM SeMet or SeOMet for a further 2 days. To evaluate the efficiency of Se translocation, Se distribution in shoot (%) was measured as the proportion of Se allocated to rice shoots. Interestingly, we found that whereas the uptake of Se by roots was significantly affected by Se forms (*P* < 0.05) but not by S or P status (*P* > 0.05), Se distribution in shoot (%) was significantly affected by nutrient status (*P* < 0.05) but not by Se forms (*P* > 0.05). Moreover, the contents of Se in shoots were significantly affected by both Se forms (*P* < 0.05) and the nutrient status (*P* < 0.05) (Table 2).

**TABLE 2:** Effect of Se chemical forms supplied and nutrient status on the content, uptake rate, and proportion of Se allocated to rice shoot.

Treatment		Se content (μg g^–1^ DW)		Se uptake rate	Shoot-Se%	Total Se uptake (μg plant^–1^)
		Root	Shoot	(μg g^–1^ root DW)		
SeMet	Normal	97.4 ± 3.23a	20.2 ± 1.96a	140.7 ± 4.58a	30.6 ± 2.63a	190.0 ± 15.9a
	S-deficient	91.7 ± 3.87a	21.6 ± 1.51a	134.9 ± 7.54a	31.8 ± 1.00a	190.8 ± 14.2a
	P-deficient	100.6 ± 1.23a	18.4 ± 0.97a	138.2 ± 2.45a	27.2 ± 1.66a	199.4 ± 8.93a
SeOMet	Normal	95.4 ± 3.58a	16.8 ± 1.07ab	131.2 ± 4.04a	27.2 ± 1.79ab	181.5 ± 11.8a
	S-deficient	91.6 ± 4.54a	19.2 ± 1.17a	130.8 ± 5.95a	29.9 ± 1.46a	189.9 ± 15.9 a
	P-deficient	87.3 ± 1.47a	14.7 ± 0.70b	116.8 ± 2.92a	25.2 ± 0.87b	166.7 ± 6.85 a
Se treatment (A)		*P* = 0.0688	*P* = 0.0076	*P* = 0.0093	*P* = 0.0936	*P* = 0.1480
Nutrient status (B)		*P* = 0.3650	*P* = 0.0262	*P* = 0.2481	*P* = 0.0378	*P* = 0.8120
A × B		*P* = 0.1150	*P* = 0.8583	*P* = 0.2224	*P* = 0.8782	*P* = 0.3650

Data are presented as mean ± SE (*n* = 4). Different letters after values in the same column indicate significant differences among plants with different nutrient status (*P* < 0.05).

In general, the contents of Se detected in rice plants, Se uptake rate, total uptake of Se, and distribution in shoots were higher in plants administered SeMet (although not all the detected differences were significant). Furthermore, compared with the normal treatment, neither P nor S deficiencies appeared to have any substantial effects on either the uptake or translocation of Se by rice, although we did observe a non-significant 14.9% reduction in the uptake of Se as SeOMet in response to a deficiency in P (Table 2).

### Interactions between selenomethionine and selenomethionine-oxide

The results of interaction experiment revealed that Se uptake and translocation in rice were significantly affected by the interactions between SeMet and SeOMet (Table 3). We found that both root Se content and uptake in rice treated with both SeMet and SeOMet forms at the same total Se concentration (SeMet + SeOMet) were 90.9 and 92.5%, respectively, lower than those in plants exposed to SeMet only, although no significant differences were detected between SeMet + SeOMet and SeOMet treatments.

**TABLE 3:** Effect of SeMet and SeOMet interaction on the uptake and translocation of Se by rice.

Treatment	Se content (μg g^–1^ DW)		Se uptake rate (μg g^–1^ root DW h^–1^)	Shoot-Se (%)	Total Se uptake (μg plant^–1^)
	Root	Shoot			
SeMet	22.98 ± 0.53a	1.87 ± 0.33	27.70 ± 1.00a	16.9 ± 1.6	9.30 ± 0.58a
SeOMet	1.93 ± 0.13b	ND	1.93 ± 0.13b	ND	0.73 ± 0.06b
SeMet+SeOMet	2.08 ± 0.08b	ND	2.08 ± 0.08b	ND	0.75 ± 0.05b
*P*	<0.001	–	<0.001	–	<0.001
Theoretical quantity	11.9 ± 1.05	0.93 ± 0.18	14.2 ± 1.50	15.7 ± 1.55	5.01 ± 0.58

The theoretical quantity calculated for the different proportions of SeMet and SeOMet treatments is based on the actual measured Se contents in rice tissues in single-SeMet or SeOMet treatments. Data are presented as the mean ± SE (*n* = 4). Different letters after values within the same column indicate a significant difference among the treatments (*P* < 0.05).

The efficiency of Se translocation during 1 h treatments was also expressed in terms of Se distribution in shoot (%). However, we were able to detect Se only in the shoots of those plants treated with SeMet treatment, which accounted for 16.9% of the total Se. In addition, the total uptake of Se by the roots of plants exposed to SeMet was between 11.4- and 11.7-fold higher than that in the SeOMet and SeMet+SeOMet treatments (*P* < 0.001). According to the calculations described by Longchamp et al. (2013) and Wang et al. (2019), in the absence of an interaction between two Se chemical forms, the total uptake of Se by roots should theoretically be 5.01 μg in the SeMet + SeOMet treatment. However, our findings indicated a total Se uptake of only ∼15% of the theoretical quantity in plants exposed to a mixture of SeMet and SeOMet, which accordingly tends to indicate a non-additive effect. This suggests that during uptake and translocation in rice, SeMet and SeOMet may interact to a certain extent.

Data are presented as the mean ± SE (*n* = 4). Different letters after values within the same column indicate a significant difference among the treatments (*P* < 0.05).

## Discussion

### Selenium uptake kinetics in rice root

The capacity of plants to take up Se from soil depends on plant species, soil Se concentration and form, and environmental conditions (including pH, Eh, and organic matter content). A number of previous studies have reported the uptake kinetics of different Se chemical forms in plant roots. For example, Huang et al. (2015) observed that the uptake of selenite into rice roots was considerably more rapid than that of selenate, with the value of *V*_*max*_ for selenite influx (102 μg⋅g^–1^ root h^–1^ DW) being approximately 6.5-fold higher than that for selenate (13.7 μg⋅g^–1^ root h^–1^ DW), with *K*_*m*_ values of 16.2 and 11.3 μM for selenite and selenate, respectively. Similarly, Hu et al. (2018) demonstrated that, for Se uptake by wheat roots, selenite (*V*_*max*_: 25.6 μg⋅g^–1^ root h^–1^ DW) is characterized by a higher uptake potential than nanoselenium (*V*_*max*_: 10.1 μg⋅g^–1^ root h^–1^ DW). Moreover, Zhang et al. (2019) obtained a *V*_*max*_ value of 132 μg⋅g^–1^ root h^–1^ DW for the uptake of SeMet. In the present study, we found that rice are characterized by a higher uptake potential (*V*_*max*_) for organic Se than for inorganic forms, showing a descending order of SeMet > SeOMet > selenite > selenate (Figure 1 and Table 1). Similarly, Wang M. K. et al. (2020) demonstrated that the roots of maize (*Zea mays* L.) had a higher uptake of organic Se (SeMet, MeSeCys, and SeCys) than inorganic Se (selenite and selenate) when supplied with 0.01 or 1 mg⋅L^–1^ Se. Consistently, Kikkert and Berkelaar (2013) found that the rate of SeMet uptake by the wheat roots was considerably higher than that of either selenite or selenate and conjectured that this difference could be attributable to the differences in the activities of their respective transporters. Moreover, in the present study, the differences in the rates of selenite and SeOMet uptake by rice roots varied according to the Se concentration exogenously applied Se (Figure 1). Likewise, Kikkert and Berkelaar (2013) found that the rate of selenite uptake was 60% lower than that of selenate, when supplied at a Se concentration of 0.5 μM but was 3.6 times higher when supplied with 5 μM Se. Collectively, these findings tend to indicate that the Se uptake capacity of plants might also be influenced by the interaction between the Se chemical form and the Se exposure level.

Regarding the two organic Se compounds (SeMet and SeOMet), this study showed that the uptake rate of SeMet was significantly greater than that of SeOMet during the initial 26 h of treatments (Figures 1, 2), although the extent of the observed differences narrowed when measured at 48 and 72 h (Figure 2). We speculated that this pattern reflected the fact that during the latter part of the treatment period, SeMet uptake was gradually approaching a level of saturation at a decreasing rate. Additionally, the effects of exposure time on organic Se absorption could be attributed to the transformation of SeMet to SeOMet in rice roots or within the rhizosphere, a conjecture that is supported by previous findings indicating that SeOMet is detectable in the roots of lettuce (*Lactuca sativa* L.) exposed to SeMet (Kowalska et al., 2020). Similarly, in a previous study, we detected both SeMet and SeOMet in the roots of selenite-treated wheat (Li et al., 2008), thereby indicating an occurrence of the oxidative transformation of Se in plants.

### The mechanisms of selenomethionine and selenomethionine-oxide uptake

To investigate the physiological processes associated with the uptake of SeMet and SeOMet by rice and the underlying mechanisms, effects of selected inhibitors on Se uptake have been assessed in this study. Among these, CCCP is a respiratory inhibitor that promotes a dissipation of the proton motive force across membranes. We found that whereas the uptake of SeOMet is sensitive to CCCP, but unaffected on SeMet (Figure 3). These observations contrast with the findings of previous hydroponic studies, which have revealed that CCCP significantly inhibits the uptake of SeMet by rice and wheat, thereby tending to indicate that SeMet is taken up by an energy-dependent symport process (Abrams et al., 1990; Zhang et al., 2019). We suspect that the disparity between the findings of these different studies could be due to differences in the respective exposure times (only 1 h in this study), which should be confirmed by further studies. Nevertheless, our findings do indicate that SeOMet uptake is a metabolically active process requiring selective binding sites and metabolic energy.

Furthermore, the findings of our specific inhibitor treatments indicate that SeMet and SeOMet are taken up into rice roots *via* different channels. Among the other inhibitors we studied, AgNO_3_ is a potential inhibitor of aquaporins of plant origin and partially inhibit the uptake of selenite (Zhang et al., 2006) and Nano-Se (Wang M. K. et al., 2020). The mechanism of its inhibit function is that silver reacts with the sulfhydryl group of a cysteine and also with a histidine, thereby resulting in a gating of the targeted aquaporins (Niemietz and Tyerman, 2002). The other three assessed inhibitors, TEACl, CoCl_2_, and DIDS, are recognized as specific inhibitors of K^+^ (White, 1995), Ca^2+^ (Harada and Shimazaki, 2009), and anionic channels, respectively. In the present study, we found that the addition of AgNO_3_ to the uptake solution significantly reduced the rate of SeMet uptake by 93.1% (Figure 3), thereby providing evidence that rice absorbs SeMet mainly *via* aquaporins. With respect to SeOMet, we observed that both AgNO_3_ (41.8%) and TEACl (45.6%) can significantly inhibit uptake by rice roots (Figure 3), which might indicate that the influx of SeOMet is mediated *via* both aquaporins and K^+^ channels. However, exposure to CoCl_2_ and DIDS exhibited no appreciable effects on Se uptake, which would accordingly tend to indicate that the uptake of SeOMet and SeMet is associated with neither Ca^2+^ nor anion channels.

Depriving plants of S significantly increases the uptake of selenate, whereas P starvation induces significant increases in selenite uptake (Li et al., 2008). And there is a competition between selenate and sulfate for uptake by roots (de Souza Cardoso et al., 2022). In the present study, we found that neither S nor P deficiency had the effect of promoting the uptake of SeMet or SeOMet by rice roots (Table 2), which might indicate that the uptake of organic Se is independent of sulfate or phosphate transporters. Conversely, under P-deficient conditions, a slight reduction was observed in the root uptake of Se as SeOMet (Table 2). In this regard, phosphorylation is one of the factors associated with the regulation of K^+^ channel activity (Yu et al., 2006), and thus, a reduction in SeOMet uptake under P-deficient conditions is attributable to diminished K^+^ channels activity, which needs to be confirmed by further in-depth molecular studies.

### The translocation of selenomethionine and selenomethionine-oxide from roots to shoots

In rice exposed to different sources of Se for 48 h, we found that the proportions of Se distributed in the shoots of rice supplied with the two assessed organic forms of Se ranged from 25.2 to 31.8% (Table 2), which are lower than the values we previously recorded in plants supplied with selenate, although slightly higher than those in plants treated with selenite (Huang et al., 2015). In this regard, the findings of several studies on rice and wheat indicated that most of the selenate taken up by roots is subsequently translocated to the shoots (Wang et al., 2015). Conversely, having been absorbed by roots, selenite is rapidly converted to organic forms, such as SeMet, MeSeCys, and SeOMet, which reduces mobility (da Silva et al., 2020). Sulfate transporters such as Sultr2;1, Sultr3;5, and Sultr1;3, are the main transporters involved in the translocation of selenate from roots to shoots (Maruyama-Nakashita, 2017; Mehdawi et al., 2018), whereas phosphorus transporters such as OsPT8 transport selenite in plants (Song et al., 2017). Furthermore, Zhang et al. (2019) found that NRT1.1B, a member of the peptide transporter family, mediates the transport activity of SeMet, whereas in maize, Wang K. et al. (2020) recorded that the values of Se distribution in shoot (%) decreased in the order of selenate treatment > selenite treatment > SeMet treatment when supplied as 0.01 mg L^–1^ Se and in the order of selenate treatment > SeMet treatment > selenite treatment when plants were supplied with 0.1 mg L^–1^ Se. Moreover, Kowalska et al. (2020) found that the translocation of Se from the roots to leaves of lettuce supplied with SeMet was 3.65 times higher than that in lettuce supplied with selenite. These phenomena can presumably be attributed to the differing capacities of the transporters of different Se chemical forms. In the present study, no significant difference was found in the transfer factor of Se from rice roots to shoots between SeMet and SeOMet (Figure 2D and Table 2), indicating the similar transportation ability of SeMet and SeOMet in rice plants; while the lowest transfer factor at 18 h might be due to the lowest transpiration rate when treated for 18 h (5:00 am), since the transportation of micro-element from roots to shoots is mainly driven by transpiration (Van der Vliet et al., 2007). Furthermore, we also found that the contents of Se were higher in the shoots of plants supplied with SeMet than in those of plants receiving SeOMet treatment when exposed for up to 26 h (Figure 2C and Table 3), although the differences were found to gradually diminish with a prolongation of exposure (Figure 2C and Table 2). This effect of exposure time on organic Se accumulation in shoots is conceivably associated with the transformation of Se in rice plants.

In addition, we established that the uptake and translocation of Se by rice plants maintained in the growth medium treated with both SeMet + SeOMet were considerably lower than the theoretical quantities (Table 3), indicating an interaction between SeMet and SeOMet when both Se forms are supplied simultaneously. In this regard, previous studies have reported non-additive effects in the uptake and translocation of different Se chemical forms, with the coexisting selenite being found to inhibit selenate uptake and translocation (Li et al., 2008; Wang et al., 2019). Similarly, in the present study, we found that the presence of SeOMet appeared to suppress the uptake and translocation of SeMet in rice. We speculate that these observations could be explained in terms of a preferential absorption by plants, whereby an optimal absorption strategy is adopted based on intrinsic synergistic activities in response to mixed supplies of different Se chemical forms, thus conserving energy required for subsequent Se assimilation (Versini et al., 2016). However, this needs to be verified in further studies.

## Conclusion

In this study, we demonstrated that the uptake and translocation of Se by rice were significantly influenced by both Se chemical forms and treatment time. Compared with inorganic forms, organic Se exhibited a higher uptake potential over the course of a 1 h exposure, with recorded uptake capacity (*V*_*max*_) values declining in the order of SeMet > SeOMet > selenite > selenate. Furthermore, analysis of the time-dependent kinetics of organic Se uptake by roots revealed that, regardless of the duration of exposure in Se-treated growth media, the uptake of SeMet was invariably higher than that of SeOMet, whereas difference between the uptake of these two forms narrowed with time. A similar tendency was detected with respect to the Se in rice shoots. In addition, examination of the effects of selected inhibitors on Se uptake indicated that SeOMet uptake is an energy-dependent symport process and that SeOMet could be imported by rice roots *via* aquaporins and K^+^ channels. In contrast, the uptake of SeMet by roots appears to be mediated primarily *via* aquaporins. We also found that when simultaneously supplied with both SeOMet and SeMet, SeOMet appeared to inhibit the uptake and translocation of SeMet.

## Data availability statement

The original contributions presented in this study are included in the article/Supplementary material, further inquiries can be directed to the corresponding author.

## Author contributions

QW, YW, and HL designed the research and wrote the manuscript. QW performed the experiments. LK helped in data analysis. QH helped in sample determination. All authors read, reviewed, and approved the manuscript.
